# EDDS-Enhanced Phytoremediation of Cd–Zn Co-Contaminated Soil by *Sedum lineare*: Mechanisms of Metal Uptake, Soil Improvement, and Microbial Community Modulation

**DOI:** 10.3390/plants15020231

**Published:** 2026-01-12

**Authors:** Haochen Shen, Ziyi Liu, Chen Wang, Ying Chu, Chuhan Zhang, Yang Yu, Shaohui Yang

**Affiliations:** 1 School of Environmental Science and Engineering, Tianjin University, Tianjin 300354, China; 2 Teda Greening Science and Technology Group Co., Ltd., Tianjin 300457, China

**Keywords:** EDDS, *Sedum lineare*, metal bioavailability, phytoremediation, soil microbial ecology, Cd-Zn co-contaminated soil

## Abstract

Soil co-contamination with cadmium (Cd) and zinc (Zn) poses serious threats to environmental safety and public health. This study investigates the enhancement effect and underlying mechanism of the biodegradable chelator Ethylenediamine-N,N′-disuccinic acid (EDDS) on phytoremediation of Cd-Zn contaminated soil using *Sedum lineare*. The results demonstrate that EDDS application (3.65 g·L^−1^) effectively alleviated metal-induced phytotoxicity by enhancing chlorophyll synthesis, activating antioxidant enzymes (catalase and dismutase), regulating S-nitrosoglutathione reductase activity, and promoting leaf protein synthesis, thereby improving photosynthetic performance and cellular integrity. The combined treatment significantly increased the bioavailability of Cd and Zn in soil, promoted their transformation into exchangeable fraction, and resulted in removal rates of 30.8% and 28.9%, respectively. EDDS also modified the interaction patterns between heavy metals and essential nutrients, particularly the competitive relationships through selective chelation between Cd/Zn and Fe/Mn during plant uptake. Soil health was substantially improved, as evidenced by reduced electrical conductivity, enhanced cation exchange capacity, and enriched beneficial microbial communities including *Sphingomonadaceae*. Based on the observed ion antagonism during metal uptake and translocation, this study proposes a novel “Nutrient Regulation Assisted Remediation” strategy to optimize heavy metal accumulation and improve remediation efficiency through rhizosphere nutrient management. These findings confirm the EDDS–*S. lineare* system as an efficient and sustainable solution for remediation of Cd–Zn co-contaminated soils.

## 1. Introduction

Soil serves as a vital foundation for ensuring food security and ecological balance, providing critical ecosystem services such as nutrient cycling, water regulation, biodiversity maintenance, and pollution buffering [1]. However, against the backdrop of industrialization and agricultural intensification, soil heavy metal contamination has emerged as a global environmental problem. In particular, cadmium-zinc (Cd-Zn) co-contamination has attracted widespread attention due to its prevalence, low detectability, and synergistic toxicity [2]. Cd and Zn exhibit similar geochemical behaviors and often coexist in non-ferrous metal mining areas and phosphate fertilizer-contaminated soils [3]. As a leading global producer in both Zn-lead (Pb) ore and phosphate fertilizer manufacturing, China is experiencing significant regional accumulation of Cd-Zn co-contamination [4,5], which not only inhibits crop root development and photosynthesis [6] and disrupts soil microbial community structure [7,8] but also threatens human health through food chain transfer and biomagnification effects, which particularly damage the renal, skeletal, and neurological systems [9].

Phytoremediation is widely recognized as a sustainable approach for soil pollution remediation due to its low cost, environmental friendliness, and ease of implementation [10,11], which relies on the ability of hyperaccumulator plants to take up, translocate, and concentrate heavy metals, thereby remediating contaminated soil through the harvesting of plant biomass [12]. However, its practical application still faces multiple bottlenecks, including low metal bioavailability, slow plant growth, limited biomass production, and prolonged remediation duration [13,14].

Chelator-assisted phytoremediation enhances plant uptake of target metals by increasing their solubility and mobility in soil [15]. While traditional chelators such as ethylene diaminetetraacetic acid (EDTA) demonstrate notable effectiveness, their high persistence and low biodegradability pose risks of secondary pollution and potential ecological hazards [16]. In contrast, EDDS has emerged as a research focus for green remediation strategies due to its strong complexation ability, environmental compatibility, and favorable biodegradability [17,18,19]. Specifically, EDDS can form stable, water-soluble complexes with Cd^2+^ and Zn^2+^ (log K ~ 13–16), effectively enhancing their phytoavailability [18,19,20,21]. Crucially, its core advantage over persistent chelators like EDTA lies in its favorable biodegradability. After mobilizing metals, EDDS can be degraded by soil microorganisms, thereby significantly mitigating the long-term risks of metal leaching and secondary ecological pollution, aligning with the principles of green and sustainable remediation. Its efficacy in improving Cd extraction has been validated in plants such as *Solanum nigrum* [22], *Helianthus annuus* [23], and *Tagetes patula* [24]. However, existing research has predominantly focused on single-metal contamination [25]. Systematic studies on the synergistic remediation of Cd-Zn co-contaminated systems using EDDS and *S. lineare* remain scarce. Key scientific questions, such as the mechanisms of competitive absorption and translocation between metal ions (e.g., Cd/Zn or Fe/Mn), rhizospheric microecological responses, and multi-element interaction behaviors, have yet to be thoroughly elucidated [26,27,28,29].

The selection of a suitable plant partner is equally critical. *Sedum lineare* exhibits remarkable drought tolerance, attributed to its Crassulacean Acid Metabolism (CAM) photosynthetic pathway [30]. It has also shown significant tolerance to various heavy metals, such as antimony, Zn, and arsenic [31,32,33]. Under metal stress, *S. lineare* activates antioxidant defense systems (e.g., superoxide dismutase SOD, catalase CAT) to maintain cellular integrity, demonstrating significant physiological and ecological adaptability [32]. Particularly noteworthy are its unique advantages of high biomass production and easy asexual propagation [34], which provide favorable conditions for large-scale remediation of contaminated sites. Our preliminary experiments showed that *S. lineare* maintains normal growth even under high Cd stress (400 mg·kg^−1^), and earlier work confirmed its substantial Zn tolerance [33]. We therefore hypothesize that *S. lineare* may possess unique advantages in remediation of Cd-Zn co-contaminated soil.

Beyond classical antioxidant responses, plants employ sophisticated signaling networks to cope with metal stress. Nitric oxide (NO) has emerged as a key signaling molecule in plant heavy-metal responses, often accumulating under Cd exposure [35,36]. NO functions largely through post-translational modifications, most notably S-nitrosylation of cysteine residues, which regulates protein activity and stress signaling [37]. The enzyme S-nitrosoglutathione reductase (GSNOR) plays a central role in maintaining NO homeostasis and controlling overall protein S-nitrosylation levels, thereby serving as a critical regulator of nitrosative stress [37]. How a biodegradable chelator such as EDDS might interact with this NO/GSNOR signaling module to influence plant metal tolerance—particularly in a co-contamination setting—remains unexplored.

Therefore, this study investigates the comprehensive mechanisms of EDDS-enhanced *S. lineare* remediation in Cd-Zn co-contaminated soil. It focuses on plant physiological and ecological responses, heavy metal absorption-translocation balance, soil chemical speciation transformation, and microbial community structure regulation, aiming to elucidate the synergistic effects of EDDS and *S. lineare*. The findings are expected to provide novel strategies and a theoretical foundation for the green and efficient remediation of heavy metal co-contaminated soils.

## 2. Materials and Methods

### 2.1. Soil Preparation and Contamination Procedure

The potting substrate consisted of vermiculite and a German peat soil (Brand K) blended at a 1:3 volume ratio. The peat material exhibited a slightly acidic pH (6.1) and contained 582.9 mg/kg total P with 19.53 mg/kg being available. Given that the native concentrations of Cd and Zn were below the detection limit, their presence in the system is attributable exclusively to experimental spiking. A Cd stock solution (4000 mg·L^−1^, aqueous concentration) was prepared by dissolving 2.034 g of CdCl_2_·2.5H_2_O in deionized water and diluted to a final volume of 250 mL. For the Cd-contaminated soil, 250 mL of the Cd stock solution and 125 mL of deionized water were added per kilogram of soil. The mixture was thoroughly homogenized and aged for 4 weeks under ventilated conditions at room temperature. This standardized procedure, widely adopted in laboratory pot experiments, allows the spiked heavy metals to reach a relatively stable state in the soil substrate [38,39]. The nominal Cd concentration in the contaminated soil was approximately 400 mg kg^−1^ (soil dry weight basis). For the Cd-Zn co-contaminated soil, a Zn stock solution (8000 mg·L^−1^) was prepared by dissolving 8.8 g of ZnSO_4_·7H_2_O in deionized water and diluted to a final volume of 250 mL. Subsequently, 250 mL of the Cd stock solution and 125 mL of the Zn stock solution were added per kilogram of soil. After thorough mixing, the soil was similarly aged for 4 weeks. The nominal Zn concentration in the co-contaminated soil was 500 mg kg^−1^.

### 2.2. Plant Culture and EDDS Application

Uniformly grown *S. lineare* seedlings (initial height: 5–6 cm) were transplanted into experimental pots (three plants per pot) and acclimatized for 7 days under controlled greenhouse conditions (22 °C, 16/8 h light/dark cycle, photosynthetic active radiation 120 μmol m^−2^ s^−1^). Soil moisture was maintained by supplementing deionized water every three days.

EDDS application followed an optimized protocol based on [40], with modifications to account for its biodegradability [41]. The plants received four applications of EDDS solution (3.65 g·L^−1^, 20 mL per pot) at 7-day intervals. The experiment was terminated 7 days after the final EDDS application, resulting in a total experimental duration of 35 days.

### 2.3. Experimental Design

The experimental design comprised twelve treatments, categorized into soil-only groups and plant-cultivated groups. The soil treatment groups (without plants) included: CK (untreated control soil), CKE (soil with EDDS), Cd (soil with Cd), CdE (soil with Cd and EDDS), CZ (soil with Cd and Zn), and CZE (soil with Cd, Zn, and EDDS). The plant treatment groups (cultivated with *S. lineare*) included: CKS (soil with *S. lineare*), CKES (soil with *S. lineare* and EDDS), CdS (soil with Cd and *S. lineare*), CdES (soil with Cd, EDDS, and *S. lineare*), CZS (soil with Cd, Zn, and *S.lineare*), and CZES (soil with Cd, Zn, EDDS, and *S. lineare*). Each pot contained 100 g dry soil (16 cm diameter, 12 cm depth).

### 2.4. Metal Analysis in Plant and Soil

For plant total metal analysis, dried samples were ground and sieved through a 60-mesh screen. A 0.1 g aliquot of the powder was digested with 10 mL of HNO_3_/HClO_4_ (4:1, *v*/*v*), including a 12-h pre-digestion at room temperature followed by hotplate heating at 320 °C until a clear, light-yellow solution remained. After cooling, the digestate was diluted to 25 mL, filtered, and analyzed for Cd, Zn, Fe, and Mn by inductively coupled plasma mass spectrometry (ICP-MS, Agilent 7800, Santa Clara, CA, USA, bought from Agilent Technologies).

For soil total metal analysis, 0.1 g air-dried soil was digested with aqua regia on a hotplate. The digestate was then filtered and diluted to 50 mL for ICP-MS measurement. The bioavailable metals in soils were extracted with CaCl_2_-TEA-DTPA following HJ804-2016 and determined by ICP-MS.

Quality control was implemented throughout the analysis. Certified reference materials (GBW07603 for plant tissues and GBW07405 for soils) were included in each digestion batch. Method blanks and duplicate samples were processed simultaneously. The recovery rates for all analyzed elements ranged from 92% to 105%. The limits of detection (LODs) for Cd, Zn, Fe, and Mn were 0.02, 1.0, 0.5, and 0.02 mg·kg^−1^, respectively.

### 2.5. Plant Accumulation Assessment

The formulas for calculating Bioconcentration Quantity (BCQ), Bioconcentration Factor (BCF), and Translocation Factor (TF) are as follows:

(1) $$BCQ=C_{plant}\times {DW}_{plant}$$

(2) $$BCF=\frac{Cplant}{Csoil}$$

(3) $$C_{plant}=\frac{\left(C_{aboveground}\times {DW}_{aboveground}\right)+(C_{underground}\times {DW}_{underground})}{{DW}_{aboveground}+{DW}_{underground}}$$

(4) $$TF=\frac{C_{abovegroud}}{C_{underground}}$$

where DW_plant_, DW_aboveground_ and DW_underground_ represent the dry weight (kg) of the whole plant, aboveground tissues and underground tissues, respectively. C_plant_, C_aboveground_, and C_underground_ denote the metal concentrations (mg·kg^−1^) in the whole plant, aboveground tissues, and underground tissues, respectively; and C_soil_ indicates the bioavailable metal concentration (mg·kg^−1^) in soil.

### 2.6. Plant Growth and Physiological Parameters

Plants from each treatment were harvested, and their roots were rinsed with deionized water. After surface moisture removal, morphological parameters including fresh weight, root length, shoot length, maximum leaf length, and internode distance were measured. Leaf water content was determined by comparing fresh and dry weights after oven-drying at 75 °C to constant weight.

Chlorophyll content was determined using the ethanol extraction method [42]. Fresh leaf samples (0.1 g) were extracted with 95% ethanol in darkness until complete bleaching, and absorbance was measured at 665 nm and 649 nm.

For enzymatic analyses, fresh leaf samples were homogenized in appropriate buffers. GSNOR activity was measured according to [43] by monitoring NADH oxidation at 340 nm. Activities of SOD and CAT, as well as levels of H_2_O_2_ and MDA, were determined using commercial assay kits (Nanjing Jiancheng Bioengineering Institute, Nanjing, China) following the manufacturer’s protocols. Superoxide anion (O_2_^−^·) was detected visually through NBT staining. Leaf samples were incubated in 50 mM sodium phosphate buffer (pH 7.8) containing NBT for 12 h in darkness, followed by destaining with 90% ethanol to remove chlorophyll for visualization.

### 2.7. Soil Physicochemical Analysis

Soil pH was determined in a 1:2.5 (*w*/*v*) soil-to-water suspension measuring the supernatant with a pH meter. Electrical conductivity (EC) was measured in a 1:5 (*w*/*v*) soil-to-water suspension. The cation exchange capacity (CEC) was measured according to the method of Jiang et al. [44]. The available phosphorus concentration was determined following the procedure established by Liu et al. [12].

The chemical fractionation of Cd and Zn was determined using the Tessier sequential extraction procedure [45]. A 1.0 g of air-dried soil was sequentially extracted to isolate five metal fractions: (1) exchangeable (1 mol/L MgCl_2_, pH 7.0); (2) carbonate-bound (1 mol/L NaOAc, pH 5.0); (3) Fe-Mn oxide-bound (0.04 mol/L NH_2_OH·HCl in 25% HOAc, 96 ± 3 °C); (4) organic matter-bound (H_2_O_2_-NH_4_OAc digestion); and (5) residual (complete digestion). After each extraction step, supernatants were collected by centrifugation and prepared for analysis. All extracts were diluted to 50 mL with 1% HNO_3_ and concentrations of Cd and Zn were determined by ICP-MS.

### 2.8. Microbial Community Analysis

After the 35-day experimental period, rhizosphere soil samples were collected from the planted treatment. Samples from individual replicates within the same treatment were combined to form composite samples. For the unplanted control groups, bulk soil was collected from a 5 cm depth and similarly composited. The microbial community composition was analyzed by sequencing the 16S rRNA gene (e.g., V3–V4 hypervariable region) of all composite samples [46]. Data processing and analysis were performed using the BMKcloud platform (www.biocloud.net).

### 2.9. Statistical Analysis

All measurements were performed with three independent replicates, and data are presented as mean ± standard deviation (SD). Statistical analyses were conducted using SPSS 26.0. The significance of differences among treatment groups was assessed using one-way analysis of variance (ANOVA), followed by Duncan’s multiple range test for post hoc comparisons; significant differences (*p* < 0.05) are denoted by different letters or asterisks in the Figures. Data visualization, correlation analysis (based on Pearson correlation coefficients), and heatmap generation were performed using Origin 2024 (including its Correlation Plot App).

## 3. Results

### 3.1. The Physiological Responses of S. lineare Under Heavy Metal Stress

#### 3.1.1. Tolerance of *S. lineare* to Heavy Metals

**Figure 1:**
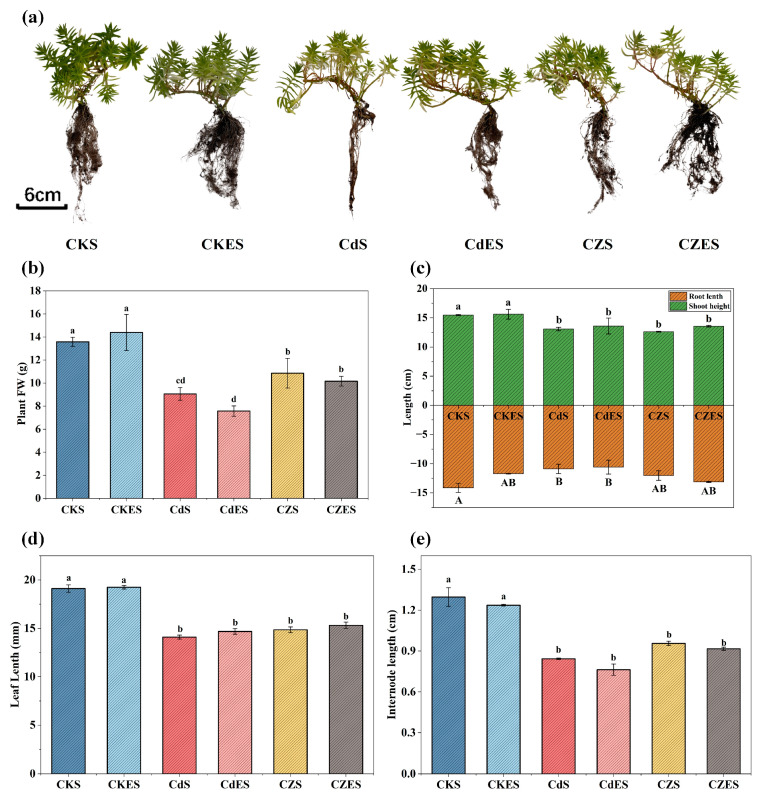
Growth parameters of *S. lineare* plants under different treatments. (**a**) Plant phenotype; (**b**) Whole-plant fresh weight; (**c**) Shoot height and root length; (**d**) Maximum leaf length; (**e**) Internode length. The above mean (±SD) was calculated from three replications. Different letters indicate statistically significant differences at *p* < 0.05.

In the present study, various growth parameters of *S. lineare* under different stress with the application of EDDS were measured. As shown in Figure 1a, *S. lineare* exhibits strong tolerance to Cd and Zn. Although extremely high concentrations of Cd (400 mg·kg^−1^) adversely affected the growth of *S. lineare*, the plant was capable of establishment and sustained growth. Compared to the control (CKS), the treatment led to decreases ranging from 15.42% to 34.88% in key growth parameters, including fresh weight, root length, plant height, leaf length, and internode distance (Figure 1b–e).

It is particularly noteworthy that exposure to 500 mg·kg^−1^ Zn alleviated the inhibitory effect of high Cd on *S. lineare*. In the CZ group, the inhibition rates of fresh weight and root length were only 20.1% and 14.95%, respectively, which were significantly lower than the 33.28% and 22.97% caused by the Cd treatment alone (Figure 1b,c). However, the mitigating effect of Zn on Cd toxicity was not statistically significant in terms of leaf length and internode length (Figure 1d,e).

#### 3.1.2. Change of Chlorophyll Content in *S. lineare*

**Figure 2:**
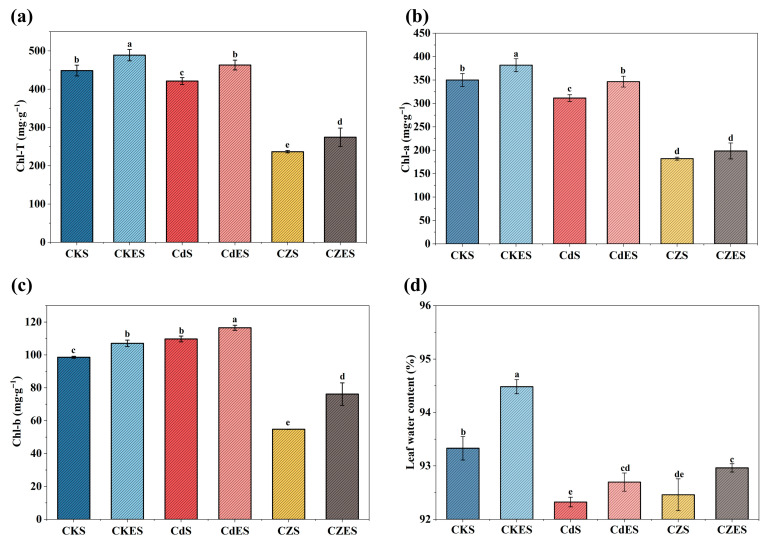
Physiological parameters of *S. lineare* under different treatments. (**a**) Total chlorophyll; (**b**) chlorophyll-a content; (**c**) chlorophyll-b content; (**d**) Leaf water content. The above mean (±SD) was calculated from three replications. Different letters indicate statistically significant differences at *p* < 0.05.

The chlorophyll content in *S. lineare* was significantly influenced by the type of heavy metal stress. Contrary to the mitigation observed in some growth parameters, Zn addition in combination with Cd (Cd-Zn group) led to a more severe suppression of chlorophyll synthesis compared to Cd stress alone. This was visually evident as exacerbated leaf chlorosis (Figure S1) and quantitatively confirmed by a significant reduction in chlorophyll a, b, and total chlorophyll content by 41.57%, 50.04%, and 43.78%, respectively, relative to the Cd-only treatment (Figure 2a). In contrast, Zn addition significantly inhibited the photosynthetic process. Specifically, the combined Cd-Zn stress further suppressed chlorophyll synthesis compared to Cd stress alone, resulting in more severe leaf chlorosis (Figure S1). The contents of chlorophyll a, b, and total chlorophyll content in the co-contamination group were further reduced by 41.58%, 50.04% and 43.78%, respectively, relative to the Cd-only treatment (Figure 2a–c).

EDDS is a biodegradable chelator with strong complexation capacity and a favorable biosafety profile [47]. In this study, application of EDDS at 3.65 g·L^−1^ did not induce phytotoxicity and even slightly increased leaf water content (Figure 2d). More notably, EDDS addition significantly alleviated the heavy metal-induced inhibition of chlorophyll synthesis, leading to a marked increase in chlorophyll content by 15.97–38.99% in the metal-stressed groups. These results support the feasibility of the combined EDDS-*S. lineare* remediation strategy.

#### 3.1.3. Change of Oxidative Stress and Antioxidant Enzyme Activities

**Figure 3:**
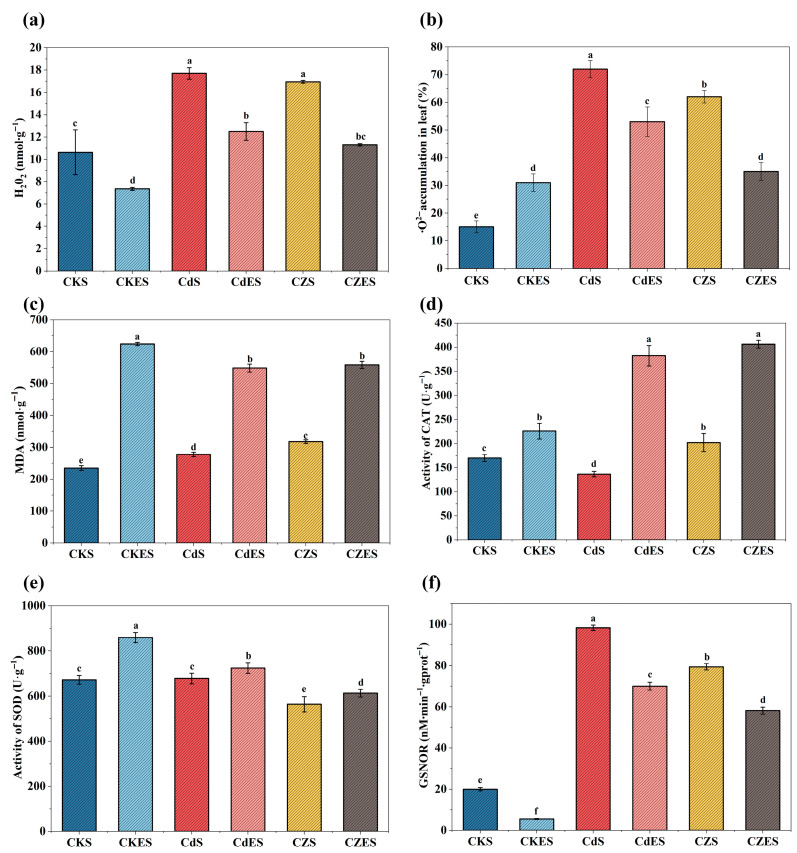
Oxidative stress and antioxidant enzyme activities in *S. lineare*. (**a**) H_2_O_2_ content; (**b**) O_2_^−^· content; (**c**) MDA content; (**d**) Activities of CAT; (**e**) Activity of SOD; (**f**) Activity of GSNOR. The above mean (±SD) was calculated from three replications. Different letters indicate statistically significant differences at *p* < 0.05.

Heavy metal stress significantly induced the accumulation of reactive oxygen species (ROS) in *S. lineare*. Compared with the control (CKS), the combined Cd-Zn stress (CZS) elevated the contents of H_2_O_2_ and O_2_^−^· in the leaves by 59.49% and 300%, respectively (Figure 3a,b), while the malondialdehyde (MDA) content increased by 35.27% (Figure 3c), indicating severe membrane lipid peroxidation damage. Interestingly, the addition of EDDS exhibited a dual effect under different stress conditions: in the CdES and CZES treatments, although it further increased MDA content, it also markedly activated the antioxidant enzyme system. Specifically, catalase (CAT) activity in CdES and CZES was significantly enhanced by 179.49% and 101.13%, respectively, compared to their EDDS-free counterparts, and superoxide dismutase (SOD) activity also showed a consistent increasing trend (Figure 3d,e). Correspondingly, EDDS treatment effectively scavenged some ROS, reducing H_2_O_2_ content in CdES and CZES by 29.38% and 33.39%, respectively, compared to their non-EDDS controls, and also significantly suppressed O_2_^−^· accumulation (Figure 3b and Figure S2). These results suggest that EDDS may partially alleviate oxidative stress by preferentially activating key antioxidant enzymes such as SOD and CAT, but it may also impose additional pressure on the cell membrane, either due to its own properties or through the process of mobilizing heavy metals.

#### 3.1.4. Change of the S-Nitrosoglutathione Reductase (GSNOR) Activity

In addition to the typical antioxidant enzyme responses, the activity of GSNOR—a key enzyme involved in regulating nitrosative homeostasis—was markedly induced under heavy metal stress, showing an approximate 3- to 4-fold increase compared to the control group (CKS) (Figure 3f). Specifically, the Cd treatment (CdS) and the combined Cd–Zn treatment (CZS) led to GSNOR activity elevations of 391.14% and 296.91%, respectively, far exceeding the responses of conventional antioxidant enzymes such as SOD and CAT.

Notably, the addition of EDDS effectively mitigated this metal-induced surge in GSNOR activity. In the CdES group, the GSNOR activity declined by 28.74% relative to the CdS group, while in the CZES group, it decreased by 26.75% compared to CZS. This consistent suppression of GSNOR highlights the important role of EDDS in regulating Cd-Zn resistance in *S. lineare*.

### 3.2. Metal Accumulation and Translocation

The capacity of plants to accumulate and translocate heavy metals is a central indicator for evaluating phytoremediation efficiency. In this study, the heavy metal content in the shoots and roots of *S. lineare* was analyzed, and the bioconcentration factor (BCF) and translocation factor (TF) were calculated to systematically evaluate the effects of EDDS and Zn-Cd interactions on the absorption and distribution patterns of heavy metals in the plant.

#### 3.2.1. EDDS Enhanced Cd Accumulation in Roots

**Figure 4:**
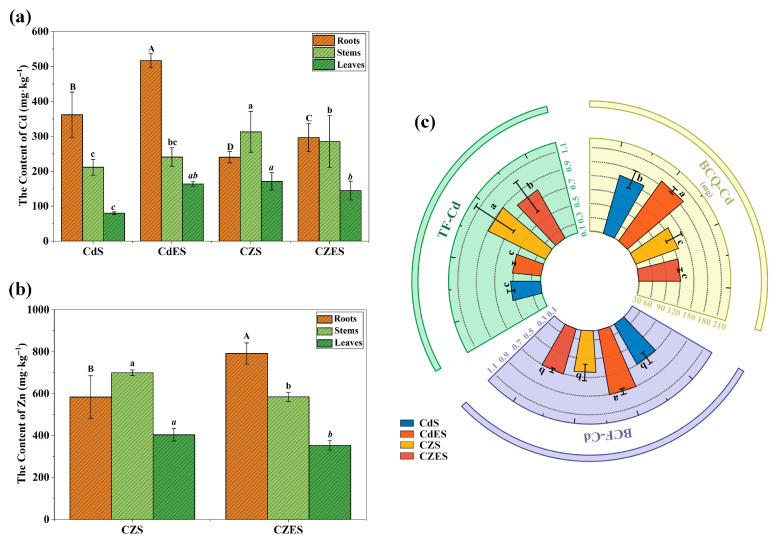
Accumulation and translocation of Cd and Zn in *S. lineare*. (**a**) Cd content; (**b**) Zn content; (**c**) BCF, BCQ and TF of Cd under different treatments. The above mean (±SD) was calculated from three replications. Different letters indicate significant differences (*p* < 0.05) among treatments within each tissue: uppercase for roots, lowercase for stems, and italic lowercase for leaves.

EDDS application significantly enhanced the Cd accumulation capacity of *S. lineare* in the root under single Cd stress. The root Cd content in the CdES treatment reached 517.45 mg·kg^−1^, representing a 42.98% increase compared to the Cd-only treatment (361.9 mg·kg^−1^) (Figure 4a). Correspondingly, the bioconcentration factor for Cd (BCF-Cd) increased from 0.61 to 0.88 (Figure 4c), a rise of 44.3%, indicating enhanced Cd enrichment in root tissues.

However, under Cd–Zn co-contamination, the promoting effect of EDDS on Cd accumulation was weakened. Although root Cd and Zn contents in the CZES group increased by 23.35% and 35.62%, respectively, compared to the CZS group, the Cd accumulation in CZES was still 43.23% lower than that in the CdES group. This suggests that the presence of high Zn levels suppressed Cd enrichment in *S. lineare*, and the facilitating effect of EDDS was partially offset by Cd–Zn competition.

#### 3.2.2. Zn Promotes Cd Translocation Under Co-Contamination While EDDS Inhibits It

The addition of Zn significantly promoted the upward translocation of Cd in *S. lineare*. As shown in Figure 4c, the translocation factor for Cd (TF-Cd) was substantially higher in the Cd-Zn co-treatment groups than in the Cd-alone groups, regardless of EDDS application. In the absence of EDDS, the TF-Cd value in the CZS group (1.1) increased by 168.42% compared to the CdS group (0.41) exceeding the hyperaccumulation threshold (1.0). With EDDS addition, the TF-Cd in the CZES group (0.75) remained significantly higher than that in the CdES group (0.39). This finding suggests that Zn facilitates the Cd transport to shoots.

Notably, under Cd-Zn co-contamination, EDDS addition partially counteracted this Zn-induced promotion of Cd translocation. The TF-Cd in the CZES group decreased by 31.67% compared to the CZS group (Figure 4c), implying that EDDS likely facilitates the retention of Cd/Zn complexes in the root tissues and thereby inhibits their upward transport.

#### 3.2.3. Competitive Uptake and Translocation of Fe/Mn and Cd/Zn

**Figure 5:**
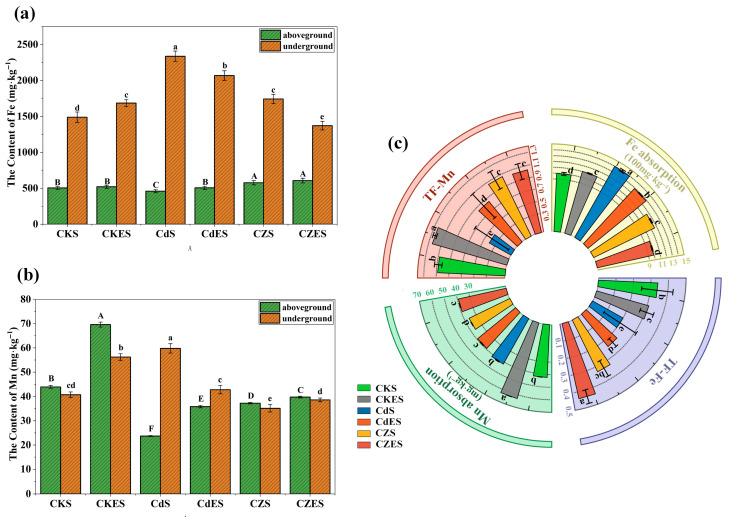
Accumulation and translocation of Fe and Mn in *S. lineare*. (**a**) Fe content in different tissues; (**b**) Mn content in different tissues; (**c**) Uptake amount and TF of Fe and Mn. The above mean (±SD) was calculated from three replications. Different letters indicate significant differences (*p* < 0.05) among treatments within each tissue: uppercase for aboveground, lowercase for underground.

**Figure 6:**
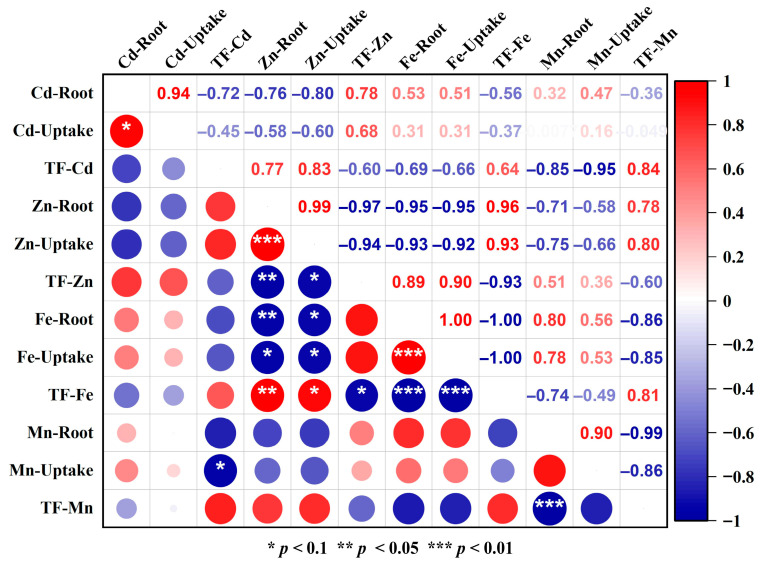
Heatmap of correlation analysis among metal uptake and translocation parameters. Red indicates a positive correlation, blue represents a negative correlation. The opacity level is related to the strength of the correlation. The white area indicates a correlation of 0.

Heavy metal stress significantly altered the uptake and translocation of essential micronutrients Fe and Mn in *S. lineare*. Under single Cd stress, root Fe and Mn contents increased significantly by 56.93% and 46.77%, respectively, compared to the control (CKS) (Figure 5a,b), indicating enhanced root uptake of these elements. However, this promotive effect was suppressed by Zn addition, resulting in 25.42% and 41.21% lower root Fe and Mn contents in the CZS group relative to the CdS group. A significant negative correlation was observed between root Zn accumulation and Fe uptake (r = −0.95), confirming competitive uptake between these elements (Figure 6).

In terms of translocation, Cd stress markedly inhibited the upward transport of both Fe and Mn to shoots. The TF-Fe decreased from 0.34 in CKS to 0.20 in CdS (a 41.18% reduction), while TF-Mn declined by 62.96% (Figure 5c). Notably, Zn addition alleviated the Cd-induced inhibition of Fe translocation, as TF-Fe in the CZS group recovered to a level comparable to the control. A similar alleviating trend was observed for Mn translocation under co-contamination. Furthermore, a strong negative correlation was observed between TF-Cd and root Mn uptake (r = −0.95, Figure 6), suggesting that Mn accumulation in roots might impede the upward transport of Cd.

This study revealed that EDDS significantly reshaped the interactions among elements. It promoted the absorption of Fe and Mn under non-stressed conditions, whereas it suppressed the uptake of both elements under Cd-alone stress. Under Cd–Zn co-contamination, EDDS intensified inter-ion competition in the rhizosphere, inhibiting Fe absorption while enhancing Mn uptake. Compared to the CZS group, the CZES group showed a 14.73% decrease in Fe content and an 8.29% increase in Mn content. These findings demonstrate that EDDS differentially regulates the absorption of distinct elements in a manner highly dependent on the ambient metal stress context.

### 3.3. Soil Remediation Efficiency

#### 3.3.1. Increased Metal Bioavailability

**Figure 7:**
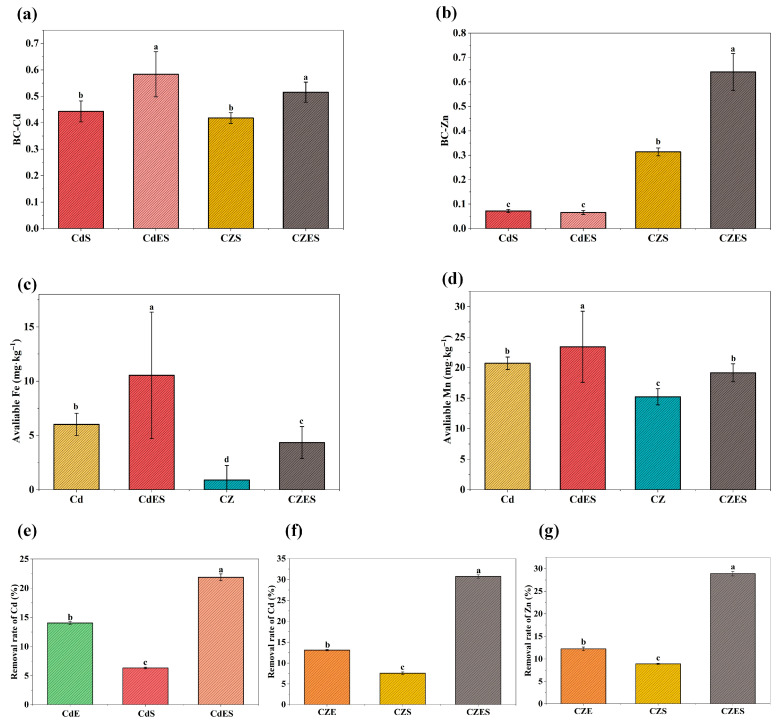
Remediation efficiency in contaminated soil. (**a**) BC-Cd; (**b**) BC-Zn. (**c**) Available Fe; (**d**) Available Mn; (**e**) Cd removal rate under Cd stress; (**f**) Cd removal rate under Cd-Zn co-stress; (**g**) Zn removal rate under Cd-Zn co-stress; The above mean (±SD) was calculated from three replications. Different letters indicate statistically significant differences at *p* < 0.05.

The combined EDDS–*S. lineare* treatment significantly enhanced the bioavailability of both Cd and Zn in contaminated soil, with a particularly pronounced effect on Zn (Figure 7a,b). The bioavailability coefficient (BC, ratio of available content to total content) results demonstrated that the BC-Cd and BC-Zn values in the CZES group under Cd-Zn co-contamination reached 0.52 and 0.64, respectively—increases of 23.81% and 106.45% compared to the CZS group (BC-Cd: 0.42, BC-Zn: 0.31). In Cd-alone contaminated soil, the BC-Cd value increased from 0.44 in the CdS group to 0.58 in the CdES group, a rise of 31.82%.

As shown in Figure 7a,b, in Cd-alone contaminated soil, the treatment increased available Fe and Mn contents by 75.08% and 13.03%, respectively. Under Cd–Zn co-contamination, the enhancement was even more substantial: available Fe content increased nearly fourfold, while available Mn content rose by 25.84%. These results indicate that the EDDS–*S. lineare* system not only effectively mobilizes target heavy metals but also differentially regulates the availability of essential elements, with a far more pronounced promoting effect on Fe than on Mn.

#### 3.3.2. Enhanced Total Removal Rates of Cd and Zn

The primary objective of phytoremediation is to reduce the total metal content in soil. In this study, the combined application of EDDS and *S. lineare* for 35 days resulted in the highest removal rates of Cd and Zn, significantly exceeding those achieved by either treatment alone. Specifically, the removal rates for Cd reached 30.75% in the CZES group under co-contamination conditions, and 21.87% in the CdES group under Cd-alone stress (Figure 7e,f). The Zn removal rate in the CZES group was 28.90% (Figure 7g). Notably, the remediation efficiency of the combined treatment significantly exceeded the sum of the individual EDDS treatment and phytoremediation alone, indicating a significant synergistic effect between EDDS and *S. lineare*.

#### 3.3.3. Transformation of Metal Speciation

**Figure 8:**
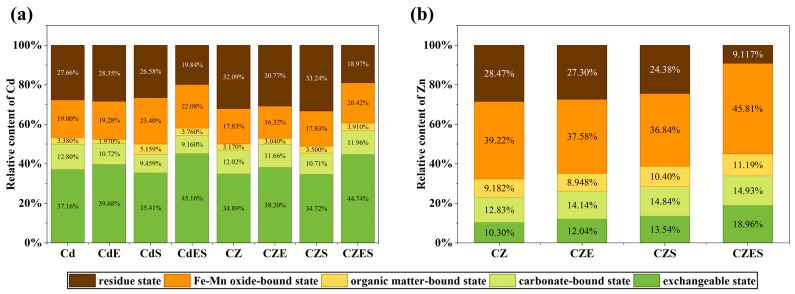
Transformation of Cd (**a**) and Zn (**b**) speciation in soil under different treatments.

The Tessier sequential extraction results demonstrated that the combined EDDS–*S. lineare* remediation remarkably altered the speciation distribution of heavy metals. For Cd (Figure 8a), the speciation distribution across all treatments followed the order: exchangeable > residual > Fe-Mn oxide bound. The combined remediation treatments (CdES, CZES) significantly promoted the transformation of Cd from stable (e.g., residual) fractions to bioavailable (e.g., exchangeable) fractions, showing the highest proportion of exchangeable Cd (CdES: 45.16%; CZES: 44.74%) and the lowest residual fraction (CdES: 19.84%; CZES: 18.97%). This indicates that the remediation process not only mobilized Cd but also facilitated its removal.

For Zn (Figure 8b), the organically-bound fraction was dominant (~40%). The combined treatment also enhanced Zn mobilization, with the CZES group exhibiting the highest proportions of exchangeable and carbonate-bound Zn and the lowest residual fraction. Notably, the combined treatment significantly increased the organically-bound Zn fraction in the CZES group (45.81%), suggesting that Zn might be fixed by root-secreted organic substances. While this likely reduces immediate biotoxicity, it might maintain potential long-term bioavailability.

#### 3.3.4. Improvement of Soil Physicochemical Properties

**Figure 9:**
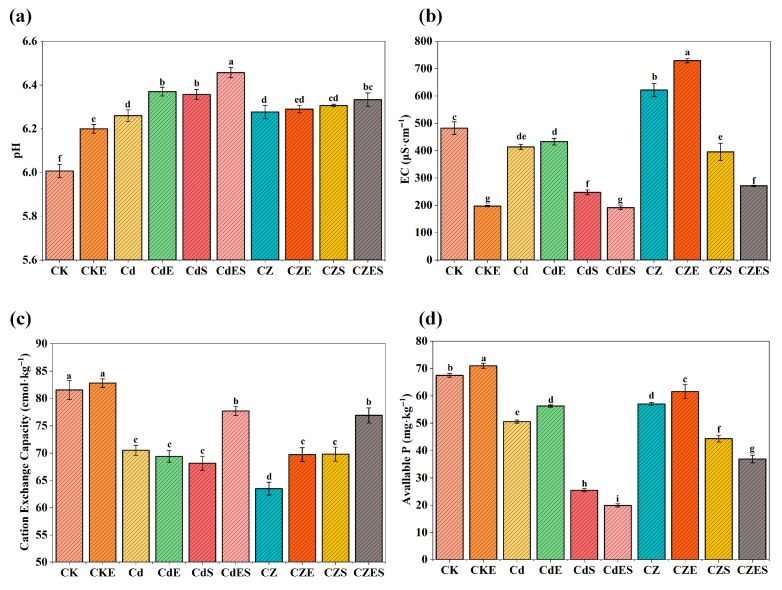
Physicochemical properties of soil under different treatments. (**a**) pH value; (**b**) Electrical conductivity (EC); (**c**) Cation exchange capacity (CEC); (**d**) Available phosphorus content. The above mean (±SD) was calculated from three replications. Different letters indicate statistically significant differences at *p* < 0.05.

The combined EDDS–*S. lineare* remediation not only facilitated metal removal but also significantly improved soil physicochemical properties. The treatment increased soil pH by 0.3–0.5 units (Figure 9a) and substantially reduced soil electrical conductivity (EC). Specifically, EC decreased by 53.75% in the CdES group and 56.35% in the CZES group relative to their corresponding contamination groups (Cd and CZ, respectively) (Figure 9b). Heavy metal stress, particularly under Cd–Zn co-contamination (CZ group), markedly suppressed soil cation exchange capacity (CEC) (Figure 9c). In contrast, the combined EDDS–plant treatment effectively restored CEC, with the CZES group showing a 20.66% increase over the CZ group, indicating enhanced soil nutrient retention and buffering capacity after remediation.

Furthermore, EDDS application alone increased the available phosphorus content. Specifically, the CKE, CdE and CZE groups showed increases of 5.16%, 11.21% and 7.98%, respectively, compared to their corresponding non-EDDS treatments. In contrast, when EDDS was combined with plant cultivation (e.g., CdES and CZES groups), the available phosphorus content decreased due to uptake and utilization by *S. lineare*. Additionally, Zn addition alleviated Cd-induced phosphorus fixation, as evidenced by the significantly higher available phosphorus content in CZ and CZE groups compared to Cd and CdE groups under both EDDS and non-EDDS conditions (Figure 9d).

### 3.4. Microbial Community Response

#### 3.4.1. Changes in Alpha Diversity

**Figure 10:**
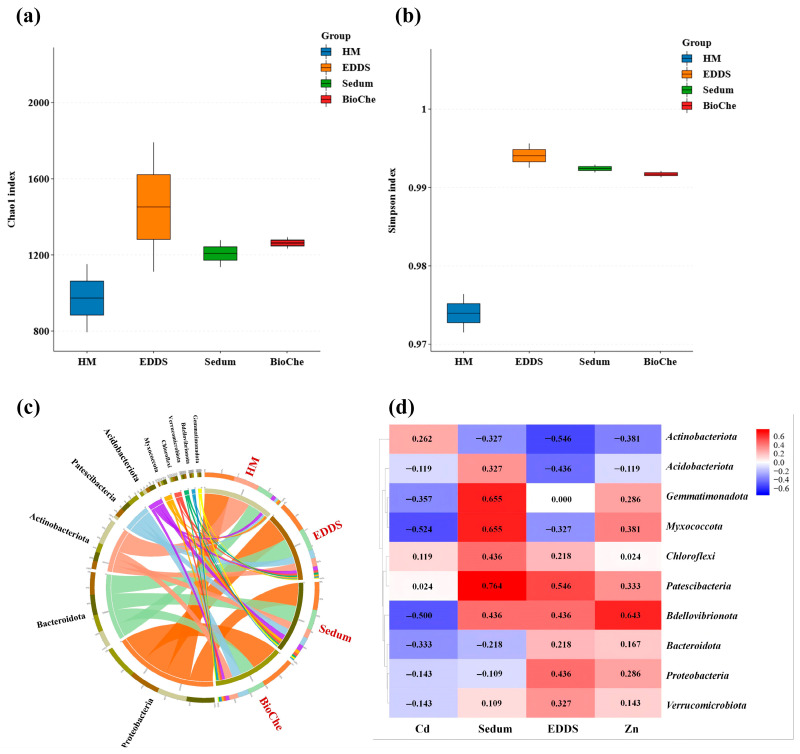
Microbial community structure and diversity in soil. (**a**) Chao1 index; (**b**) Simpson index; (**c**) Microbial composition of the top 10 phyla by abundance, to illustrate the differences among the repair methods, we grouped the different samples. “Cd, CZ” are classified as HM, “CdS, CZS” are classified as Sedum, “CdE, CZE” are classified as “EDDS”, “CdES, CZES” are classified as “BioChe”; (**d**) Heatmap of correlation analysis of microbial enrichment across different treatments.

Heavy metal stress (HM group) significantly reduced soil microbial species richness. All remediation treatments—EDDS alone, *S. lineare* alone, and their combination—increased the Chao1 index compared to the HM group, with the combined EDDS–*S. lineare* treatment (BioChe group) showing the most pronounced recovery (Figure 10a). The Simpson index was significantly higher in all remediation groups than in the HM group (Figure 10b), though no significant differences were observed among remediation treatments.

#### 3.4.2. Changes in β Diversity

Principal Coordinate Analysis indicated distinct shifts in soil microbial community structure across treatments (Figure S3). The HM group formed a distinct cluster separate from all remediation treatments (Figure S3a), confirming heavy metals as the primary disruptor of soil microbial structure. Both EDDS application and *S. lineare* cultivation significantly reshaped communities (Figure S3b,c). EDDS induced more pronounced shifts, reflecting its direct chemical disturbance, whereas plant-driven changes were more consistent.

#### 3.4.3. Shifts in Microbial Community Structure

Remediation treatments significantly altered soil microbial community composition. At the phylum level, all remediation groups showed increased relative abundance of *Chloroflexi*, *Gemmatimonadota*, and *Myxococcota* compared to the HM group (Figure 10c). Correlation analysis (Figure 10d) quantified the relationships between environmental factors and microbial phyla. Soil Cd content was the primary negative factor suppressing microbial diversity, showing strong negative correlations with most phyla, particularly *Myxococcota* and *Bdellovibrionota*. In contrast, the cultivation of *S. lineare* served as the core positive driver, being positively correlated with the enrichment of beneficial taxa such as *Gemmatimonadota*, *Myxococcota*, and *Patescibacteria*. EDDS application exhibited a more specific regulatory effect, primarily associated with the increase in *Patescibacteria* abundance. Notabely, soil Zn content showed a relatively limited overall impact, exhibiting a significant positive correlation only with *Bdellovibrionota*.

**Figure 11:**
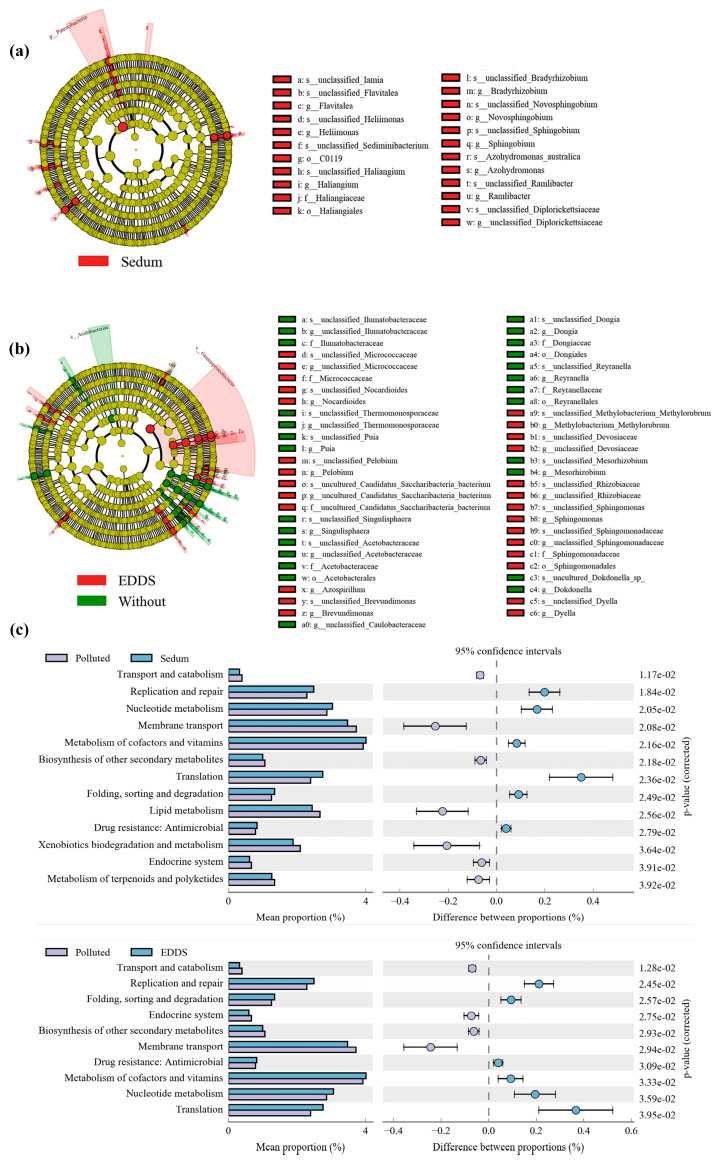
Functional and taxonomic analysis of soil microbial community. (**a**) LefSe analysis of *S. lineare* group, in order to analyze the impact of *S. lineare* planting on soil microorganisms, we combined CdS, CZS, CdES, and CZES into “Sedum”; (**b**) LefSe analysis of EDDS-treated group, in order to analyze the impact of EDDS on soil microorganisms, we combined CdS and CZS as “without”, and combined CdE, CZE, CdES, CZES as “EDDS”; (**c**) KEGG functional annotation.

Further LefSe analysis indicated that *S. lineare* rhizosphere effect significantly enriched *Patescibacteria* and *Bdellovibrionota* (Figure 11a), while EDDS treatment specifically enriched *Sphingomonadaceae* (Figure 11b).

#### 3.4.4. Functional Shifts Revealed by KEGG Pathway Prediction

KEGG functional prediction of the microbial community showed distinct metabolic profile changes among treatments (Figure 11c). Compared to the heavy metal contamination group (HM), both EDDS treatment and *S. lineare* cultivation increased the relative abundance of genes related to genetic information processing (including replication and repair, translation, nucleotide metabolism) and metabolism of cofactor and vitamins.

The remediation groups also exhibited higher abundance in pathways involved in xenobiotic biodegradation and metabolism, and drug resistance (e.g., antibiotic resistance). In contrast, the HM group showed higher relative abundance of functions associated with membrane transport and the endocrine system.

## 4. Discussion

As a biodegradable alternative to traditional chelators, EDDS exhibits comparable metal complexation capacity while minimizing long-term environmental risks due to its rapid microbial degradation [48]. This study demonstrates that the combined EDDS–*S. lineare* system not only enhances the removal of Cd and Zn from co-contaminated soil but also improves soil physicochemical properties and microbial ecological functions, achieving the dual goals of pollution remediation and soil health restoration.

### 4.1. Synergistic Enhancement of Phytoremediation Efficiency

The superior performance of the EDDS–*S. lineare* system stems from a synergistic dual enhancement: EDDS significantly increases heavy metal bioavailability, while concurrently improving plant physiological tolerance. This creates a self-sustaining “Mobilization-Uptake” cycle that continuously transfers metals from soil to plant biomass.

Mechanistically, EDDS forms stable, water-soluble complexes with Cd^2+^ and Zn^2+^, displacing them from soil adsorption sites and enhancing their solubility [18]. Sequential extraction confirmed a dramatic redistribution of metal pools: the combined treatment promoted the transformation of Cd from stable residual fractions to bioavailable exchangeable forms (e.g., exchangeable Cd increased to ~45% in CdES and CZES groups), indicating EDDS actively “unlocks” previously fixed metals. For Zn, the treatment increased organically-bound fractions (~45.81% in CZES group; Figure 8b), likely facilitated by interactions with root exudates, highlighting the regulatory role of the rhizosphere in metal speciation.

Concurrently, EDDS alleviated metal phytotoxicity, enabling *S. lineare* to maintain robust growth. This mitigation was directly evidenced by a significant recovery (15–40%; Figure 2) in chlorophyll content under metal stress, reflecting the protection of photosynthetic function. Such recovery is critical, as heavy metals like Cd are known to directly impair photosynthesis by inhibiting chlorophyll biosynthesis and inactivating key enzymes [49]. At a physiological level, EDDS activated key antioxidant enzymes (SOD and CAT), reduced oxidative stress markers (H_2_O_2_, O_2_^−^·). Notably, while heavy metal stress significantly upregulated the activity of GSNOR (391.14% in the CdS group)—a key enzyme in managing nitrosative stress—EDDS treatment substantially suppressed this induction (28.74% reduction in the CdES group, Figure 3f). This suggests that EDDS intervenes in the NO-mediated stress signaling network. We hypothesize that EDDS fine-tunes NO signaling, potentially attenuating the S-nitrosylation of key functional proteins like phytochelatins (PCs), thereby preserving their metal-chelating capacity under stress. This finding offers a novel perspective on the post-translational modification mechanisms underpinning heavy metal tolerance in plants. Thus, the EDDS-driven mobilization and plant-based uptake/sequestration form a positive feedback loop that maximizes remediation efficiency.

The Cd accumulation capacity of *S. lineare* observed in this study (e.g., up to 537 mg/kg in roots) is comparable to or exceeds that reported for other remediation plants such as *Lonicera japonica* (70 mg/kg) and *Crassocephalum crepidioides* (120 mg/kg) [50,51]. Importantly, its rapid growth and high biomass yield translate into a significant advantage in total metal removal capacity—a critical metric for practical remediation [34]. However, the key advancement of this work extends beyond documenting plant accumulation. It lies in establishing a synergistic remediation system with the biodegradable chelator EDDS, which not only enhanced metal bioavailability and plant tolerance but also drove concurrent recovery of soil health and ecological function, moving toward a more holistic remediation paradigm.

### 4.2. Interplay Between Heavy Metals and Essential Nutrients

This study reveals complex interactions between heavy metals and essential nutrients during their uptake and translocation in *S. lineare*. At the root uptake level, the competition for divalent metal transporters—notably the ZIP and NRAMP families—creates a critical interface where metal species compete for entry [52]. For instance, IRT1, a well-characterized member of the ZIP family in *Arabidopsis thaliana*, is known to transport Fe, Cd, Mn, and Zn, illustrating the inherent potential for these ions to compete for the same uptake pathways [53]. The significant suppression of Fe uptake by Zn (r = −0.95, Figure 6) provides direct evidence for this competitive exclusion mechanism. Under Cd stress alone, the increased root Fe and Mn contents (56.93% and 46.77%, respectively; Figure 5) might result from either Cd^2+^-induced membrane damage facilitating non-selective ion influx or compensatory upregulation of nutrient uptake systems in response to functional deficiency [54].

Within the plant, translocation processes reveal further regulation: Cd strongly inhibited the upward transport of Fe and Mn, likely by interfering with xylem loading or competing for internal chelators like nicotianamine [52]. Conversely, Zn promoted Cd translocation (TF-Cd increased by ~168% in CZS group), attributable to their chemical similarity and shared transport pathways [55,56]. However, under co-contamination, EDDS partially inhibited Cd translocation (~32% reduction in CZES group), suggesting EDDS-metal complexes may be preferentially retained in roots via sequestration mechanisms.

EDDS acts as a context-dependent modulator, leveraging its distinct complexation strengths (log K: Fe^3+^ > Zn^2+^ > Cd^2+^ > Mn^2+^) [20,21]. It differentially regulates metal interactions: enhancing Fe/Mn uptake under non-stress conditions while, under Cd/Zn stress, EDDS-Cd complexes may dominate transport pathways. These insights validate a “Nutrient Regulation-Assisted Remediation” strategy, where essential nutrients can be strategically managed based on their antagonistic/synergistic relationships with contaminants. EDDS serves as an “intelligent switch” in this strategy, enabling precise regulation through selective chelation.

### 4.3. Integrated Recovery of Soil Health and Microbial Ecology

Beyond metal removal, the EDDS–*S. lineare* system significantly improved soil health. Key physicochemical properties were enhanced: pH increased moderately (0.3–0.5 units), EC substantially decreased, and CEC was restored (Figure 9). This improvement likely results from EDDS complexation of acidifying ions, combined with root-driven improvements in soil structure.

Crucially, this pH shift did not compromise Cd phytoavailability. Although pH elevation typically reduces free Cd^2+^ bioavailability—and optimal Cd uptake without chelators is reported near pH 5–6 [57]—the formation of soluble EDDS-Cd complexes in this study effectively compensated for the decrease in free Cd^2+^, ensuring continuous plant uptake. Consequently, the moderate pH adjustment to a mildly acidic range (pH 5.6–6.4) created a more favorable soil environment for plant growth and microbial activity without sacrificing remediation efficiency.

The system also positively influenced nutrient dynamics. EDDS application alone increased soil available P (e.g., by 13.07% in CdE group, Figure 8a) by chelating metal cations (e.g., Ca^2+^, Al^3+^, Fe^3+^) that typically fix phosphorus in insoluble forms [58]. In combined treatments, however, available P decreased due to active uptake by *S. lineare*, reflecting the system’s ability to improve nutrient utilization and thereby support plant growth and remediation efficiency. Furthermore, Zn addition alleviated Cd-induced P fixation, evidenced by significantly higher available P in Cd–Zn co-contaminated groups (CZ and CZE) compared to Cd-alone groups (Cd and CdE). This may result from competition between Zn and Cd for soil sorption sites or Zn-mediated shifts in microbial/root activities, underscoring the potential of leveraging metal interactions to improve P availability in co-contaminated soils.

EDDS also mobilized essential micronutrients: it significantly increased soil-available Fe and Mn (Figure 7c,d), while *S. lineare* exhibited substantial uptake and translocation of both elements (Figure 5a,b). The active plant uptake served as a competitive sink, buffering their net accumulation in the soil solution and thereby mitigating potential leaching risks.

Microbial community analysis confirmed the ecological compatibility of the combined strategy. Alpha diversity recovered across all remediation treatments, with the greatest restoration under the combined approach. Treatment-specific enrichment patterns emerged: *S. lineare* favored stress-resistant and predatory bacteria (e.g., *Patescibacteria*, *Bdellovibrionota*), reflecting rhizosphere-driven selection [59], while EDDS enriched metal-tolerant degraders (e.g., *Sphingomonadaceae*) known for their degradation capabilities [60]. Beta diversity indicated that plant cultivation was the primary driver of community restructuring, with EDDS providing auxiliary selective pressure. Correlation analysis (Figure 10d) further elucidated the distinct roles of key environmental drivers: soil Cd content was the primary negative factor suppressing diversity, plant cultivation served as the core positive driver restoring it, and EDDS specifically modulated certain microbial groups.

Functionally, KEGG pathway analysis showed a transition from a stress-tolerating “survival” mode to a metabolically active “growth” state, with enhanced activity in genetic processing, cofactor metabolism, and xenobiotic degradation pathways. This reflects a recovery of microbial functionality and resilience under reduced metal stress. It should be noted that this study represents an endpoint assessment after the 35-day remediation period and does not capture dynamic successional processes. Future time-series studies, coupled with targeted molecular approaches such as quantitative PCR (qPCR) for key functional genes (e.g., the *omcB* gene of dissimilatory iron-reducing bacteria such as *Geobacter* spp.) [61], are needed to precisely quantify the dynamics of critical functional taxa and establish direct links with heavy-metal speciation data. Furthermore, systematic characterization of root exudates will help clarify rhizosphere chemical-microbial interactions.

## 5. Conclusions

This study establishes an integrated and sustainable remediation strategy by synergistically combining the biodegradable chelator EDDS with the tolerant plant *S*. *lineare* for Cd–Zn co-contaminated soil. The system operated through three core mechanisms: (1) EDDS bolstered plant tolerance by activating antioxidant defenses (SOD, CAT), fine-tuning stress-signaling via GSNOR regulation, and improving chlorophyll synthesis and cellular integrity; (2) EDDS significantly enhanced the bioavailability of Cd and Zn by converting stable fractions into exchangeable forms, driving efficient plant uptake (e.g., root Cd up to 537 mg/kg) and achieving notable removal rates (30.75% for Cd, 28.90% for Zn) with a total bioconcentration quantity (BCQ-Cd) of 199.24 mg; (3) the treatment induced comprehensive soil health recovery, marked by improved physicochemical properties (reduced EC, increased CEC and pH), enhanced nutrient cycling, and the reconstruction of beneficial and functionally robust microbial communities. Importantly, the competitive interactions between Cd/Zn and essential nutrients (Fe/Mn) uncovered here underpin a novel “Nutrient Regulation-Assisted Remediation” strategy, offering a precise means to modulate metal accumulation.

The synergistic mechanisms elucidated provide a critical theoretical basis for optimizing chelant-assisted phytoremediation. Given the full biodegradability of EDDS and the strong environmental adaptability of *S. lineare*, this system holds broad application prospects for green and sustainable remediation in harsh environments such as mining areas. Future work should focus on validating these mechanisms at the molecular level (e.g., via functional gene quantification) and scaling up the strategy through field trials to assess its practical applicability and long-term ecological stability.

## Data Availability

The data presented in this study are available on request from the corresponding author. The data are not publicly available due to privacy and ethical restrictions.

## Supplementary Materials

The following supporting information can be downloaded at: https://www.mdpi.com/article/10.3390/plants15020231/s1, Figure S1. Phenotype of *S. lineare* plants under different treatments. Figure S2. Localization of superoxide anion (O_2_^−•^) by NBT staining in *S. lineare*. Figure S3. Effects of different remediation programs on soil β diversity. (A) Among Treatments; (B) EDDS vs. Without; (C) Sedum vs. Without.

## References

[1] Ding Z., Liu K., Grunwald S., Smith P., Ciais P., Wang B., Wadoux A.M.J., Ferreira C., Karunaratne S., Shurpali N. (2025). Advancing soil organic carbon prediction: A comprehensive review of technologies, AI, process-based and hybrid modelling approaches. Adv. Sci..

[2] Yu B., Miao X., Ouyang S. (2025). Soil heavy metal pollution trends for intensive vegetable production system in Beijing-Tianjin-Hebei region, China (2000–2024) and human health implications. Environ. Res..

[3] Zeb M., Khan K., Younas M., Farooqi A., Cao X., Kavil Y.N., Alelyani S.S., Alkasbi M.M., Al-Sehemi A.G. (2024). A review of heavy metals pollution in riverine sediment from various Asian and European countries: Distribution, sources, and environmental risk. Mar. Pollut. Bull..

[4] Hou S., Zheng N., Tang L., Ji X., Li Y., Hua X. (2019). Pollution characteristics, sources, and health risk assessment of human exposure to Cu, Zn, Cd and Pb pollution in urban street dust across China between 2009 and 2018. Environ. Int..

[5] Cheng X., Wei C., Ke X., Pan J., Wei G., Chen Y., Wei C., Li F., Preis S. (2022). Nationwide review of heavy metals in municipal sludge wastewater treatment plants in China: Sources, composition, accumulation and risk assessment. J. Hazard. Mater..

[6] Sirgedaitė-Šėžienė V., Striganavičiūtė G., Šilanskienė M., Kniuipytė I., Praspaliauskas M., Vaškevičienė I., Lemanas E., Vaitiekūnaitė D. (2025). Evaluating *Populus tremula* L. and *Salix caprea* L. for phytoremediation: Growth, metal uptake, and biochemical responses under arsenic, cadmium, and lead stress. Front. Plant Sci..

[7] Zeng X., Liu Y., Wang Q., Ma H., Li X., Wang Q., Yang Q. (2024). Tanning wastewater restructured nitrogen-transforming bacteria communities and promoted N_2_O emissions in receiving river riparian sediments. Environ. Res..

[8] Cao L., Xie H., Sun R., He L., Dai Z., Li C. (2025). Microplastics and heavy metals reshape mangrove rhizosphere microbiomes and compromise carbon fixation potential. Ecotoxicol. Environ. Saf..

[9] Gao Y., Weng W., Huang K., Ren S., Jordan R.W., Jiang S.J., Ji Y., Gu Y.G. (2025). Foodborne metal(loid) contamination from coastal petrochemical industrial zone to countryside and urban zones: Spatial distribution and public health implications. J. Hazard. Mater..

[10] Chen C., Bongers F.J., Schmid B., Ma K., Liu X. (2025). Ecosystem consequences of functional diversity in forests and implications for restoration. New Phytol..

[11] Igwe A.N., Callwood K.A., Shelton D.S. (2025). Restoring landscapes and communities: Insights from critical, urban, and plant ecology. Environ. Sci. Ecotechnol..

[12] Liu Z., Wen J., Liu Z., Wei H., Zhang J. (2024). Polyethylene microplastics alter soil microbial community assembly and ecosystem multifunctionality. Environ. Int..

[13] Jha A., Barsola B., Pathania D., Raizada P., Thakur P., Singh P., Rustagi S., Khosla A., Chaudhary V. (2024). Nano-biogenic heavy metals adsorptive remediation for enhanced soil health and sustainable agricultural production. Environ. Res..

[14] Dai S., Feng W., Song F., Li T., Tao Y., Yang F., Miao Q., Duan P., Liao H., Shi H. (2025). Review of biological algal fertilizer technology: Alleviating salinization, sequestering carbon, and improving crop productivity. Bioresour. Technol..

[15] Yang S., Tu C., Liu G.M., Li Y., Wang Y., Zhu X., Si S.C., Luo R.L., Li Z.Y., Luo Y.M. (2025). Waste biomass-derived organic matter for sustainable soil remediation: Enhancing heavy metal removal and eluent reuse in agricultural application. Bioresour. Technol..

[16] Hasan M.M., Uddin M.N., Ara-Sharmeen I., Alharby H.F., Alzahrani Y., Hakeem K.R., Zhang L. (2019). Assisting phytoremediation of heavy metals using chemical amendments. Plants.

[17] Bucheli-Witschel M., Egli T. (2001). Environmental fate and microbial degradation of aminopolycarboxylic acids. FEMS Microbiol. Rev..

[18] Chien S.C., Wang H.H., Chen Y.M., Wang M.K., Liu C.C. (2021). Removal of heavy metals from contaminated paddy soils using chemical reductants coupled with dissolved organic carbon solutions. J. Hazard. Mater..

[19] Tandy S., Bossart K., Mueller R., Ritschel J., Hauser L., Schulin R., Nowack B. (2004). Extraction of Heavy Metals from Soils Using Biodegradable Chelating Agents. Environ. Sci. Technol..

[20] Hauser L., Tandy S., Schulin R., Nowack B. (2005). Column Extraction of Heavy Metals from Soils Using the Biodegradable Chelating Agent EDDS. Environ. Sci. Technol..

[21] Drozd A., Ju Y., Kolodynska D. (2023). Improved Soil Amendment by Integrating Metal Complexes and Biodegradable Complexing Agents in Superabsorbents. Materials.

[22] Sharma P., Rathee S., Ahmad M., Raina R., Batish D.R., Singh H.P. (2023). Comparison of synthetic and organic biodegradable chelants in augmenting cadmium phytoextraction in *Solanum nigrum*. Int. J. Phytoremediat..

[23] Xu Z., Pan J., Ullah N., Duan Y., Hao R., Li J., Huang Q., Xu L. (2023). 5-Aminolevulinic acid mitigates the chromium-induced changes in *Helianthus annuus* L. as revealed by plant defense system enhancement. Plant Physiol. Biochem..

[24] Wang Y., Xu Y., Qin X., Liang X., Huang Q., Peng Y. (2020). Effects of EDDS on the Cd uptake and growth of *Tagetes patula* L. and *Phytolacca americana* L. in Cd-contaminated alkaline soil in northern China. Environ. Sci. Pollut. Res. Int..

[25] Beiyuan J., Fang L., Chen H., Li M., Liu D., Wang Y. (2021). Nitrogen of EDDS enhanced removal of potentially toxic elements and attenuated their oxidative stress in a phytoextraction process. Environ. Pollut..

[26] McDougall D.R., Kihara S., Reinhardt J., Miskelly G.M., McGillivray D.J., Jeffs A.G. (2020). Biodegradable chelating agent improves the survival of early larvae for shellfish aquaculture. Aquat. Toxicol..

[27] Kołodyńska D., Drozd A., Ju Y. (2021). Superabsorbents and their application for heavy metal Ion removal in the presence of EDDS. Polymers.

[28] Wang Y., Xu Y., Sun G., Liang X., Sun Y., Wang L., Huang Q. (2021). Comparative effects of *Tagetes patula* L. extraction, mercapto-palygorskite immobilisation, and the combination thereof on Cd accumulation by wheat in Cd-contaminated soil. Ecotoxicol. Environ. Saf..

[29] Zhou M., Kiamarsi Z., Han R., Kafi M., Lutts S. (2023). Effect of NaCl and EDDS on heavy metal accumulation in *Kosteletzkya pentacarpos* in polymetallic polluted soil. Plants.

[30] Shan Q., Liu X., Zhang J., Chen G., Liu S., Zhang P., Wang Y. (2011). Analysis on the tolerance of four ecotype plants against copper stress in soil. Procedia Environ. Sci..

[31] Ning Z., Xiao T., Xiao E. (2015). Antimony in the soil-plant system in an Sb mining/smelting area of southwest China. Int. J. Phytoremediat..

[32] Yang S., Yin R., Wang C., Wang J. (2023). Improved efficiency of *Sedum lineare* (*Crassulaceae*) in remediation of arsenic-contaminated soil by phosphate-dissolving strain *P-1* in association with phosphate rock. Environ. Geochem. Health.

[33] Zhang Y., Liao H. (2021). Epibrassinolide improves the growth performance of *Sedum lineare* upon Zn stress through boosting antioxidative capacities. PLoS ONE.

[34] Xu C., Zhang Q., Yan Y., Fu D. (2025). Exogenous abscisic acid reduces water consumption in *Sedum lineare* for green roofs: Insights from morpho-physio-biochemical responses and multi-omics. Plant Physiol. Biochem..

[35] Leng Y., Li Y., Wen Y., Zhao H., Wang Q., Li S.W. (2020). Transcriptome analysis provides molecular evidences for growth and adaptation of plant roots in cadimium-contaminated environments. Ecotoxicol. Environ. Saf..

[36] Luo P., Wu J., Li T.-T., Shi P., Ma Q., Di D.-W. (2024). An Overview of the Mechanisms through Which Plants Regulate ROS Homeostasis under Cadmium Stress. Antioxidants.

[37] Zhang L., Li Y., Wang Y., Liu Z., Kronzucker H.J., Wang Z., Shi W., Li G. (2025). Ion toxicity in waterlogged soils: Mechanisms of root response and adaptive strategies. Front. Plant Sci..

[38] Bandara T., Franks A., Xu J., Chathurika J., Tang C. (2021). Biochar aging alters the bioavailability of cadmium and microbial activity in acid contaminated soils. J. Hazard. Mater..

[39] Dong S., Li L., Chen W., Chen Z., Wang Y., Wang S. (2024). Evaluation of heavy metal speciation distribution in soil and the accumulation characteristics in wild plants: A study on naturally aged abandoned farmland adjacent to tailings. Sci. Total Environ..

[40] Zhang H., Zhang K., Duan Y., Sun X., Lin L., An Q., Altaf M.M., Zhu Z., Liu F., Jiao Y. (2024). Effect of EDDS on the rhizosphere ecology and microbial regulation of the Cd-Cr contaminated soil remediation using king grass combined with *Piriformospora indica*. J. Hazard. Mater..

[41] Tandy S., Ammann A., Schulin R., Nowack B. (2006). Biodegradation and speciation of residual SS-ethylenediaminedisuccinic acid (EDDS) in soil solution left after soil washing. Environ. Pollut..

[42] Zhang J., Wang S., Bai Z., Pei J., Yang S., Wang J. (2025). Overexpression of *E. coli* formaldehyde metabolic genes pleiotropically promotes *Arabidopsis thaliana* growth by regulating redox homeostasis. J. Hazard. Mater..

[43] Wang S., Zhang B., Zhang S., Yang S., Lu M.Z., Wang J. (2025). The overexpression of *E. coli* formaldehyde metabolism genes in *Arabidopsis* conferred varying degrees of resistance to oxidative stress induced by small organic compounds. J. Hazard. Mater..

[44] Jiang J., Wang Y.-P., Yu M., Cao N., Yan J. (2018). Soil organic matter is important for acid buffering and reducing aluminum leaching from acidic forest soils. Chem. Geol..

[45] Tessier A., Campbell P.G.C., Bisson M. (1979). Sequential extraction procedure for the speciation of particulate trace metals. Anal. Chem..

[46] Han N., Peng X., Zhang T., Qiang Y., Li X., Zhang W. (2020). A new quantitative 16S rRNA amplicon sequencing method. Sheng Wu Gong Cheng Xue Bao.

[47] Riyazuddin R., Nisha N., Ejaz B., Khan M.I.R., Kumar M., Ramteke P.W., Gupta R. (2021). A comprehensive review on the heavy metal toxicity and sequestration in plants. Biomolecules.

[48] Jomova K., Alomar S.Y., Nepovimova E., Kuca K., Valko M. (2025). Heavy metals: Toxicity and human health effects. Arch. Toxicol..

[49] Kosolsaksakul P., Farmer J.G., Oliver I.W., Graham M.C. (2014). Geochemical associations and availability of cadmium (Cd) in a paddy field system, northwestern Thailand. Environ. Pollut..

[50] Xie M., Gao X., Zhang S., Fu X., Le Y., Wang L. (2023). Cadmium stimulated cooperation between bacterial endophytes and plant intrinsic detoxification mechanism in *Lonicera japonica* thunb. Chemosphere.

[51] Zhu G., Xiao H., Guo Q., Zhang Z., Zhao J., Yang D. (2018). Effects of cadmium stress on growth and amino acid metabolism in two Compositae plants. Ecotoxicol. Environ. Saf..

[52] Panda A., Fatnani D., Parida A.K. (2025). Uptake, impact, adaptive mechanisms, and phytoremediation of heavy metals by plants: Role of transporters in heavy metal sequestration. Plant Physiol. Biochem..

[53] Spielmann J., Leonhardt N., Neveu J., Vert G. (2025). Canonical tyrosine-based motifs are required for constitutive endocytosis and polarity of IRT1 and contribute to metal uptake. Plant J..

[54] Cheng Y., Bao Y., Chen X., Yao Q., Wang C., Chai S., Zeng J., Fan X., Kang H., Sha L. (2020). Different nitrogen forms differentially affect Cd uptake and accumulation in dwarf Polish wheat (*Triticum polonicum* L.) seedlings. J. Hazard. Mater..

[55] Yang X.E., Long X.X., Ye H.B., He Z.L., Calvert D.V., Stoffella P.J. (2004). Cadmium tolerance and hyperaccumulation in a new Zn-hyperaccumulating plant species (*Sedum alfredii Hance*). Plant Soil.

[56] Zhou Z., Zhang B., Liu H., Liang X., Ma W., Shi Z., Yang S. (2019). Zinc effects on cadmium toxicity in two wheat varieties (*Triticum aestivum* L.) differing in grain cadmium accumulation. Ecotoxicol. Environ. Saf..

[57] Wu G., Islam M.S., Fu Q., Liu Y., Zhu J., Fang L., Hu H. (2025). Impact of citric acid on cadmium immobilization in soil amended with biochar. J. Environ. Sci..

[58] Fatima F., Pathak N., Srivastava D., Verma S.R. (2020). Molecular Detection and Exploration of Diversity Among Fungal Consortium Involved in Phosphate Solubilization. Geomicrobiol. J..

[59] Zheng Z., Li X., Huang S., Wang X., Jia X., Wang H., Zhou J., Ma L. (2025). Novel insights into microbial strategies for antimony (Sb) transformation coupled with carbon utilization in groundwater ecosystem. Environ. Int..

[60] Xiao Y., Dong M., Yang B., Wang S., Liang S., Liu D., Zhang H. (2024). Strengthening bioremediation potential: *Enterobacter ludwigii* ES2 for combined nicosulfuron and Cd contamination through whole genome and microbial diversity community analysis. J. Hazard. Mater..

[61] Liu Y., Fredrickson J.K., Zachara J.M., Shi L. (2015). Direct involvement of *ombB*, *omaB*, and *omcB* genes in extracellular reduction of Fe(III) by *Geobacter sulfurreducens* PCA. Front. Microbiol..

