# Supplementary material for: EDDS-Enhanced Phytoremediation of Cd–Zn Co-Contaminated Soil by Sedum lineare: Mechanisms of Metal Uptake, Soil Improvement, and Microbial Community Modulation
Source: Plants (Basel). 2026 Jan 12;15(2):231. doi: 10.3390/plants15020231 (PMC12845202; doi:10.3390/plants15020231)
Supplement: Supplementary file 1 [file plants-15-00231-s001.zip › plants-4054833-supplementary.pdf]

SUPPLEMENTARY MATERIALS

EDDS-enhanced phytoremediation of Cd–Zn co-contaminated soil by *Sedum lineare*: Mechanisms of metal uptake, soil improvement, and microbial community modulation

Haochen Shen[1, 2, 3], Ziyi Liu[1, 3], Chen Wang[1], Ying Chu[1], Chuhan Zhang[1], Yang Yu[1], Shaohui Yang[1]*

[1]School of Environmental Science and Engineering, Tianjin University, Tianjin 300354, China

[2]Teda Greening Science and Technology Group Co., LTD., Tianjin 300457, China

[3]These authors contributed equally to this article.

*Corresponding author. E-mail: shaohuiyang77@tju.edu.cn

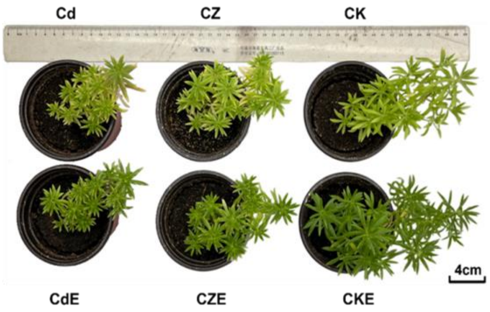

Fig. S1 Phenotype of *S. lineare* plants under different treatments.

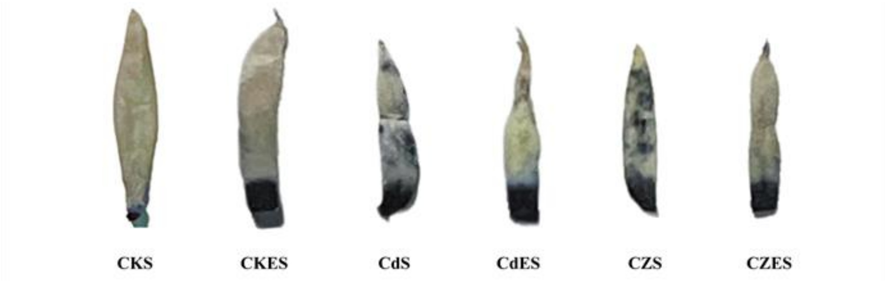

Fig. S2. Localization of superoxide anion ($O_2^{-}\cdot$) by NBT staining in *S. lineare*.

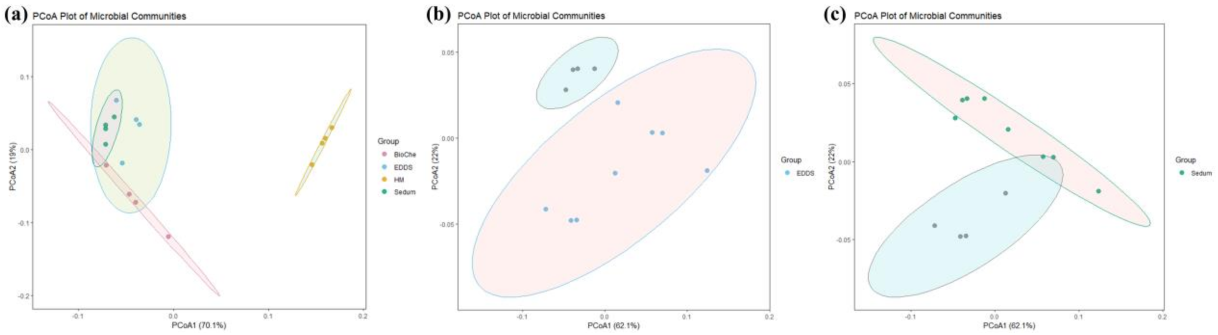

Fig. S3. Effects of different remediation programs on soil β diversity. (**a**) Among Treatments; (**b**) EDDS vs. Without; (**c**) Sedum vs. Without.
